# A Role of Activators for Efficient CO_2_ Affinity on Polyacrylonitrile-Based Porous Carbon Materials

**DOI:** 10.3389/fchem.2020.00710

**Published:** 2020-08-21

**Authors:** Urooj Kamran, Jang Rak Choi, Soo-Jin Park

**Affiliations:** ^1^Department of Chemistry, Inha University, Incheon, South Korea; ^2^Evertech Enterprise Co. Ltd., Hwaseong, South Korea

**Keywords:** porous carbons, activating agents, CO_2_/N_2_ selectivity, regenerability, CO_2_ adsorption

## Abstract

Herein, we investigated polyacrylonitrile (PAN)-based porous activated carbon sorbents as an efficient candidate for CO_2_ capture. In this research, an easy and an economical method of chemical activation and carbonization was used to generate activated PAN precursor (PAN-C) adsorbents. The influence of various activators including NaOH, KOH, K_2_CO_3_, and KNO_3_ on the textural features of PAN-C and their CO_2_ adsorption performance under different temperatures was examined. Among the investigated adsorbents, PANC-NaOH and PANC-KOH exhibited high specific surface areas (2,012 and 3,072 m^2^ g^−1^), with high microporosity (0.82 and 1.15 cm^3^ g^−1^) and large amounts of carbon and nitrogen moieties. The PAN-C activated with NaOH and KOH showed maximum CO_2_ uptakes of 257 and 246 mg g^−1^ at 273 K and 163 and 155 mg g^−1^ at 298 K, 1 bar, respectively, which was much higher as compared to the inactivated PAN-C precursor (8.9 mg g^−1^ at 273 K and 1 bar). The heat of adsorption (*Q*_st_) was in the range 10.81–39.26 kJ mol^−1^, indicating the physisorption nature of the CO_2_ adsorption process. The PAN-C-based activated adsorbents demonstrated good regeneration ability over repeated adsorption cycles. The current study offers a facile two-step fabrication method to generate efficient activated porous carbon materials from inexpensive and readily available PAN for use as CO_2_ adsorbents in environmental applications.

**Graphical Abstract:**
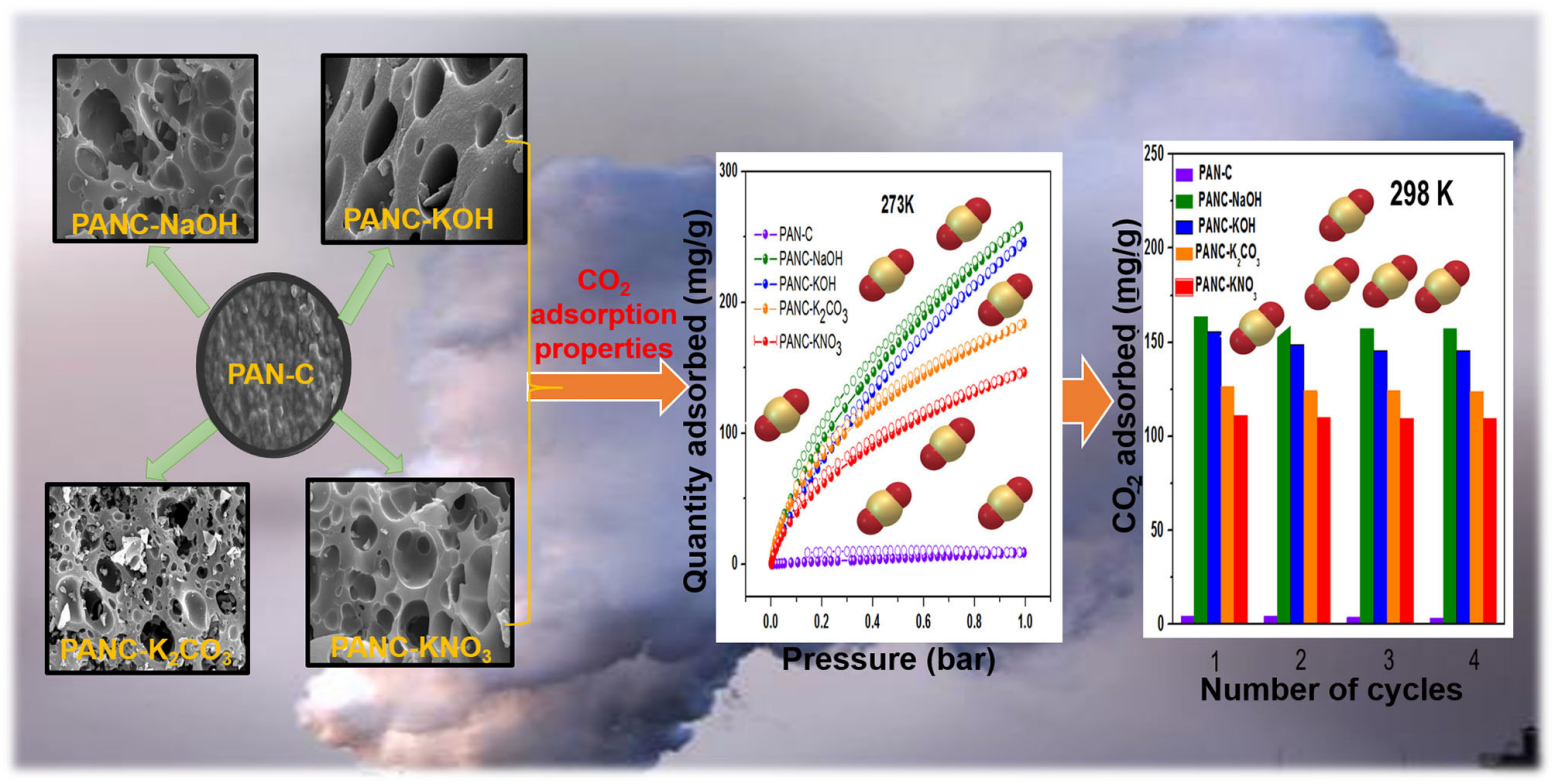
PAN-based various porous activated carbons for efficient CO_2_ adsorption performance.

## Introduction

In recent centuries, the frequency and strength of climate events such as rainstorms, cold streaks, and heat waves have prominently increased worldwide. The major critical problem due to climate variation is the continuous increase in global warming (Petrescu et al., 2017), which is responsible for increasing the overall world temperature and for sea acidification (Zhong et al., 2018). The main cause of global warming is the emission of carbon dioxide (CO_2_) (both emissions from natural sources and from anthropogenic activities such as burning of fossil fuels) into the atmosphere (Al-Mamoori et al., 2017). Therefore, mitigation of CO_2_ discharge is a forthcoming mission. Different technologies for CO_2_ capture include membrane separation, ionic liquid adsorption and amine scrubbing. The amine solution sorption technique is a conventional method but has several disadvantages, including corrosivity of the amine solution and greater power utilization for material regeneration than other methods (Rao and Rubin, 2002). The manufacture of advanced adsorbents for effective CO_2_ uptake is critical (MacDowell et al., 2010). Solid materials for adsorption-based CO_2_ capture, such as amine-modified mesoporous adsorbents (Chen et al., 2013; Sim et al., 2015), silica (Belmabkhout et al., 2009), microporous carbon-based materials (Wickramaratne and Jaroniec, 2013; Kamran and Park, 2020), zeolites (Bae et al., 2013), inorganic-capillary membranes (Besser et al., 2016), porous hexagonal boron nitride (h-BN) sheets (Kamran et al., 2019a), and nitrogen-doped carbon adsorbents (Heo and Park, 2015), are being investigated as promising alternatives because of their cost-effective preparation, broad availability, high specific surface area, controllable surface properties, physiochemical sustainability, and minimal energy utilization. Among these adsorbents, porous carbon sorbents, commonly referred to as activated carbons, have demonstrated several advantages and are considered efficient adsorbents for gas uptake, metal recovery, and catalysis (Lee et al., 2006; Liang et al., 2014; Kamran et al., 2019b), because of their high adsorptive capacity, economical processing, easy regeneration, high thermal sustainability, rapid adsorption kinetics, high specific surface area, adjustable porosity, and functionality and low sensitivity to moisture (Zhou et al., 2013). In particular, the extent of porosity induction depends on several factors, including the nature of the precursor, the activation methodology and the activation conditions.

Usually, the fabrication of porous carbon-based sorbents is followed by physical or chemical activation (Ahmadpour and Do, 1996; Park and Jang, 2002; Nowrouzi et al., 2018). Chemical activation is broadly used because, unlike physical activation, it does not require high temperatures, is rapid in nature and generates a large number of pores within the material (Das et al., 2015). However, the choice of activator is an important factor influencing the process efficacy of chemical activation. A literature survey reveals that different chemicals can be used as activating agents, including potassium hydroxide (KOH) (Wang and Kaskel, 2012), sodium hydroxide (NaOH) (Xu et al., 2010), zinc chloride (ZnCl_2_) (Meng and Park, 2014), sodium amide (NaNH_2_), potassium carbonate (K_2_CO_3_) (Hayashi et al., 2002), and potassium nitrate (KNO_3_) (Li et al., 2019a). There are the following reasons available for the selection of specific activators. KOH is broadly used because KOH activation of carbon-based materials causes the formation of large specific surface area and generation of very narrow sized microspores above carbon surface that responsible for the enhancement in CO_2_ uptakes (González-García, 2018). K_2_CO_3_ is also utilized as an activator during chemical activation of varieties of carbonaceous materials, because K_2_CO_3_ has been likely to play an essential part in the generation of highly porous morphology within the carbons, which lead to the improvement in CO_2_ adsorption capacity (Guo et al., 2015). During chemical activation with K_2_CO_3_ activator, the metallic potassium (K) inserts within the carbon framework, and K removal causes the pores and holes generation within carbon material (Viswanathan et al., 2009). For pre-oxidation of polyacrylonitrile (PAN), KNO_3_ was applied as an activator (Li et al., 2019a). The PAN activation with KNO_3_ increases the burning of combustible materials and responsible for the formation of soft and semi-carbonaceous networks for easy availability of other activating agents. As a result, ultra-high porous morphology obtained (Li et al., 2019b). Besides this, NaOH activation of carbon materials seemed more effectual, economical, and eco-friendly. Because NaOH is capable of producing extensive disordered carbon network, that was obtained by surface reaction during the chemical activation process. Resultantly, highly porous carbon materials were obtained with a large specific area (Viswanathan et al., 2009; Tan et al., 2014).

Furthermore, the chemical activation approach shows several merits, including decreased pre-oxidation temperatures, a high production rate and low costs, and it leads to a carbon framework with a high surface area and large number of pores by disordering the surface through the activation reaction, leading to activated carbons with a strong CO_2_ adsorption tendency (Viswanathan et al., 2009; Tan et al., 2014). Various nitrogen-enriched materials have been used as precursors to generate carbon-based materials, including polyaniline (Zhang X. et al., 2017), PAN (Wang et al., 2014), and aminopyrine (Zhang E. et al., 2017). Among these, PAN has received intensive attention because of its efficient advantages of having a high nitrogen content, a controllable morphology and a relatively greater carbon production rate (Shu et al., 2017). PAN is a readily available, inexpensive material with high regeneration ability, good chemical resistance, good thermal stability, low flammability, and excellent mechanical features (Saeed et al., 2008; Tahaei et al., 2008; Heo et al., 2019). Moreover, porous carbon synthesized from PAN exhibits a high carbon ratio (Ko et al., 1992), and high molecular weight (Hou et al., 2006).

In the present work, we investigated the facile fabrication of highly microporous and mesoporous carbon sorbents through carbonization of inexpensive, non-toxic, easily available polymer PAN followed by chemical activation with different types of activators/activating agents (KOH, NaOH, K_2_CO_3_, and KNO_3_). The main objective is to study the comparative influence of each activating agent on the CO_2_ adsorption capacities of PAN-based carbon (PAN-C) material. By varying the kind of activator, we tuned the textural properties (specific surface area and porosity) of the obtained PAN-C materials. The effects of the carbon, nitrogen, and oxygen contents, a high surface area and the presence of different functional groups on the CO_2_ adsorption capacity of PAN-C material were also investigated. Furthermore, by comparing activating agents, we observed that the NaOH-activated sorbents exhibited the highest CO_2_ adsorption capacity among the investigated activated carbons (257.55 mg g^−1^ at 273 K and 1 bar).

## Experimental

### Materials

PAN (average molecular weight: 150,000), zinc chloride (98%), potassium carbonate (99%), and sodium hydroxide were obtained from Sigma-Aldrich. Potassium hydroxide was purchased from Duksan Pure Chemical Co., and potassium nitrate (99%) and hydrochloric acid (35–37%) were purchased from Samchun Co. Carbon dioxide gas (99.99%) used in CO_2_ adsorption experiments was obtained from Sigma Gases. All the materials were used without further purification.

### Synthesis Protocol

**Figure 1:**
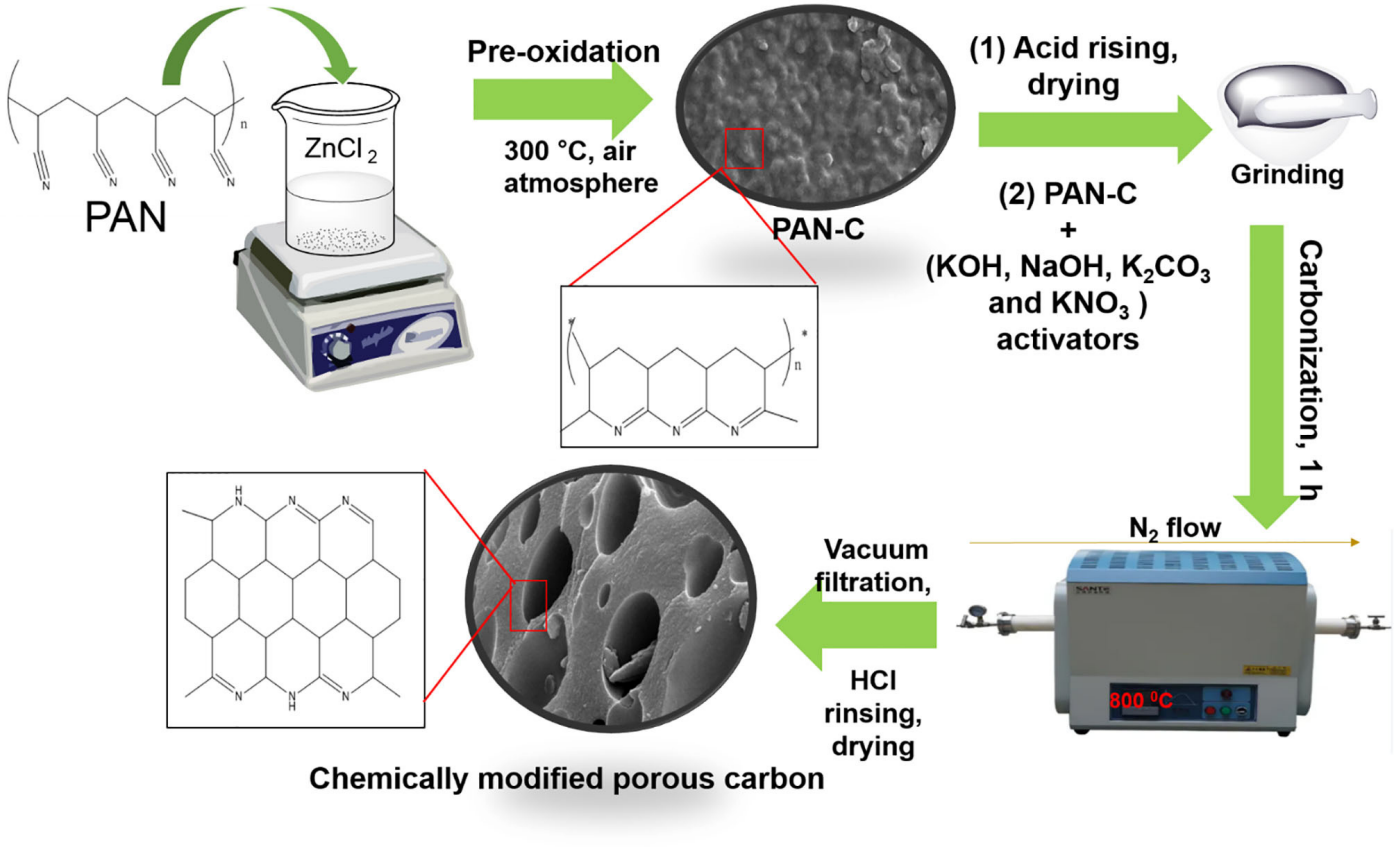
Schematic protocol with proposed mechanism from PAN to activated porous carbon adsorbents.

The PAN-based porous carbon material was fabricated as follows. Five grams of PAN was added to 250 mL of ZnCl_2_ (25 wt%). This solution was stirred for 7 h at 30°C. The obtained white precipitates were collected by suction filtration and dried at 80°C overnight. It has been examined that PAN is soluble in extremely concentrated acidic solutions as well as in concentrated inorganic salt solutions (ZnCl_2_, ZnBr_2_) because of powerful nitrile-nitrile interaction (Iovleva et al., 2001). Hence, Stoy et al. (1990) elaborated the homogeneous acidic hydrolysis method to dissolve PAN in acidic solution (specifically in concentrated ZnCl_2_ and in HNO_3_ solution). As the dissolution of PAN in 25 wt% of ZnCl_2_ was performed at a low temperature of 30°C, which lead to the generation of several irregularly distributed amide groups. These generated amidic groups merged with the PAN through co-polymerization. Furthermore, in concentrated ZnCl_2_ solution, all the nitrile functional groups in PAN are equally available to undergo solvation, regardless of the existence of irregular linkages within the polymer chain (Stoy et al., 1990). The last stage is the stabilization of cyclic framework which was obtained by cyclization of PAN usually at low temperature through pre-oxidization (Houtz, 1950). The obtained white powder was converted into a PAN precursor (PAN-C) by thermal pre-oxidation treatment in a furnace at 300°C for 4 h under air atmosphere. The ZnCl_2_ in this reaction acted as a pore creation agent that promote activation method by initiating the physical dehydration and other role of ZnCl_2_ is to form polymer complex by linking the PAN building blocks at low temperature 300°C (Sahin et al., 2002; Ashourirad et al., 2018) (as mentioned in Figure 1). The obtained PAN-C precursor were rinsing with 0.5 M HCl in order to remove the trapped ZnCl_2_ impurities inside the sample (Feng et al., 2018).

The chemical modification of PAN-C precursor with several activating agents (AAs) (including KOH, NaOH, KNO_3_, and K_2_CO_3_) was carried out by mixing the PAN-C precursor and various AAs individually in a (1:0.8) ratio with a pestle and mortar to obtain a homogeneous powder. The mixture was carbonized at 800°C for 1 h under a N_2_ atmosphere flowing at 20 mL min^−1^ and at heating rate of 10°C min^−1^. The prepared chemically modified PAN-C products were finally rinsed with 0.5 M HCl and distilled water several times until a pH of 7 was obtained to eliminate excess activating agents; the products were then dried at 120°C for 10 h in an oven. A brief outline of the method and the proposed mechanism is shown in Figure 1. The PAN-C precursor modified with different activating agents (i.e., KOH, NaOH, KNO_3_, and K_2_CO_3_) are represented as PANC-KOH, PANC-NaOH, PANC-KNO_3_, and PANC-K_2_CO_3_, respectively.

### Characterization Methods

The prepared PAN-based modified porous carbon materials were characterized by the following analytical techniques. The structural morphology of samples was obtained by high-resolution scanning electron microscopy (HR-SEM; SU8010, Hitachi Co., Japan, operated at 1 kV). Functional groups on the materials were characterized by Fourier transform infrared vacuum spectrometry (FTIR, Vertex-80 V, Bruker, USA) over the 4,000–400 cm^−1^ range. Raman spectra were recorded with a Sentera R200-L dispersive Raman microscope (Bruker Optics Co. Ltd., Germany). The thermal stability was analyzed by thermogravimetric analysis (TGA; TG-209F3, NETZSCH, Germany) under an air atmosphere in the temperature range of 25–800°C with heating rate of 10°C/min. The X-ray diffraction (XRD) peaks were obtained using an X-ray diffractometer (D-2 Phaser, Bruker, Netherlands). An elemental analyzer (EA1112, ThermoFisher Scientific, South Korea) was used to determine the elemental content of the materials. Additional surface characterization was carried out by X-ray photoelectron spectroscopy (XPS, K-α, VG Scientific Co., Waltham, MA, USA).

### Gas Adsorption Analyses

The N_2_ adsorption–desorption isotherms were measured at 77 K using a Belsorp Max (BEL-Japan) to record textural properties of samples. Prior to gas-adsorption measurements, the samples were degassed at 200°C for 6 h under vacuum to eliminate moisture and gas molecules adsorbed inside the pores of materials. The specific surface area (SSA), pore volume, diameter and pore-size distribution (PSD) were calculated using the non-local density functional theory equation (NLDFT). The CO_2_ gas adsorption experiments were performed with the Belsorp Max system (BEL-Japan Inc.) at 1 bar and at different temperatures (273, 283, and 298 K). To evaluate the gas selectivity of adsorbents; N_2_ adsorption plots were also obtained at 298 and 273 K using the same instrument.

## Results and Discussion

### Morphological and Structural Properties

**Figure 2:**
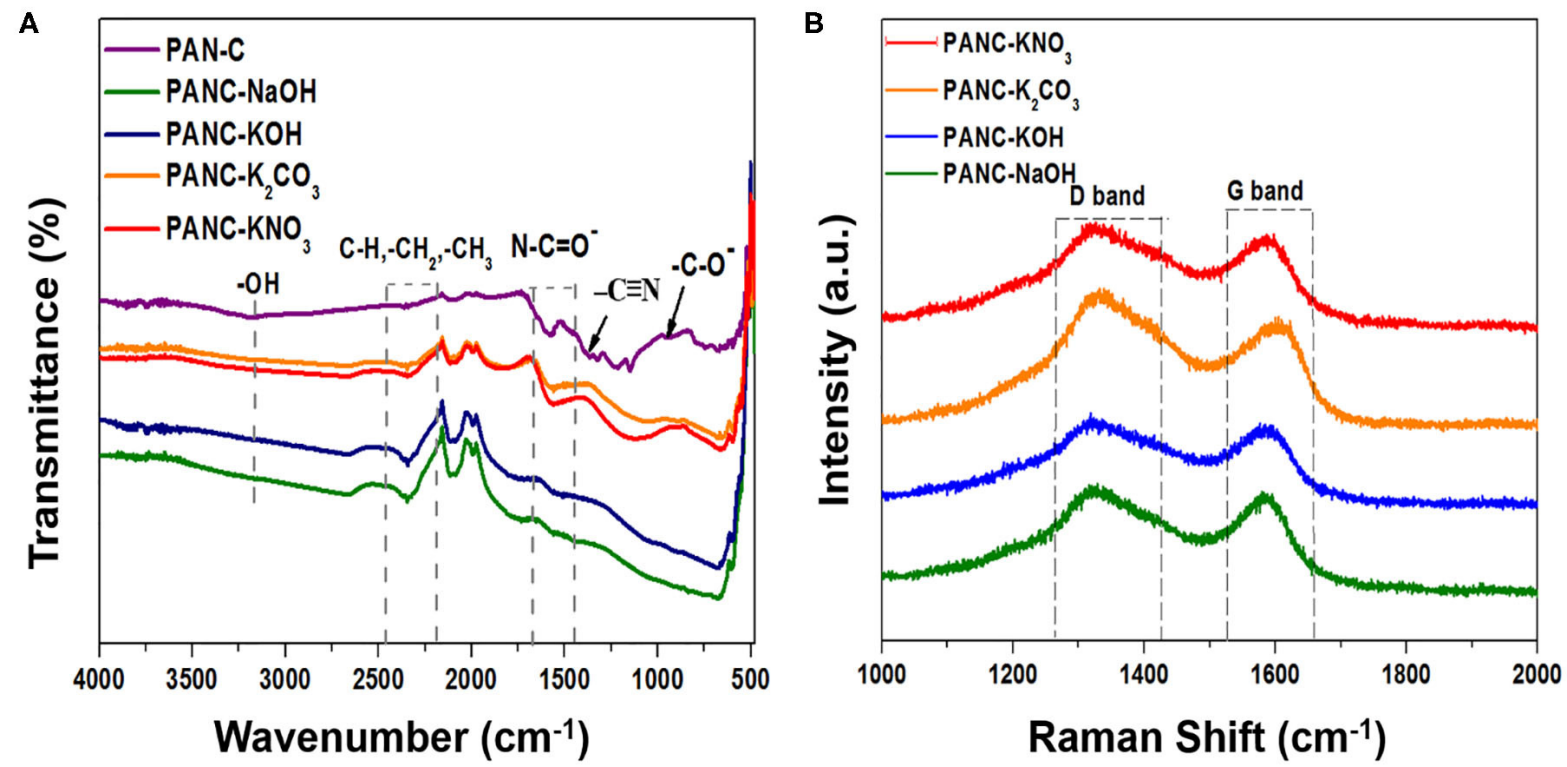
**(A)** FTIR spectra of PAN-C precursor and of all fabricated activated carbon materials and **(B)** Raman spectra of all activated carbon materials.

The functional-group analysis of the prepared samples was conducted by FTIR spectroscopy. As shown in the inset of Figure 2A, the spectrum of the PAN-C precursor (inset) shows a large shoulder at 3,171 cm^−1^, which is attributed to vibrations of hydroxyl (–OH) groups. The weak band at 2,343 cm^−1^ represents aliphatic groups (–CH, –CH_2_, and –CH_3_). The peaks at 1,589 and 1,368 cm^−1^ are associated with stretching vibrations of nitrile groups (N–C=O– and –C=N) (Dalton et al., 1999; Wangxi et al., 2003; Xue et al., 2013), and the small weak band at 942 cm^−1^ is attributed to the stretching of carbonyl (–C–O–) groups. The introduction of various activating agents and chemical activation at targeted temperature (800°C) within PAN-C precursor results in disappearance of weak nitrile bonds, which confirms the successive carbonization of PAN-C precursor. Resultantly, the presence of remaining weak nitrogen groups attributed essential role in increasing CO_2_ uptakes capacities (Xia et al., 2011). The presence of negligible weak signals of N–C=O– and –C–O– groups in the spectra of the activated samples is ascribed to the destruction of functional groups due to the reaction between precursor and activating agents during the chemical activation process (Zhang et al., 2016). Similarly, the removal of broad –OH group bands in activated carbon adsorbents confirmed the neutralization of the –OH group with activators during chemical activation.

Raman analysis was conducted to determine the degree of graphitization of the fabricated adsorbents (Figure 2B). The Raman spectra shows two distinct peaks: the peaks at ~1,320–1,331 cm^−1^ are attributed the characteristics of D-band, and those in the range 1,584–1,607 cm^−1^ are assigned to the G-band (**Table 2**). These peaks are associated with ordered graphitic structures and disordered graphitic structures with defects, respectively. The comparative ratio between the G-band intensity (IG) and D-band intensity (ID) indicates the nature of the graphitic material as well as the degree of graphitization. The determined ID/IG ratio of all activated adsorbents are 1.03 (for PANC-NaOH), 1.04 (for PANC-KOH), 0.96 (for PANC-K_2_CO_3_), 1.02 (for PANC-KNO_3_); the results are summarized in **Table 2**. The greater ID/IG values showing the generation of more disordered carbon framework and poor crystallinity (Vázquez-Santos et al., 2012). According to ID/IG values, the PANC-NaOH and PANC-KOH adsorbents exhibit highly amorphous and disordered structure with a greater degree of graphitization as compared to PANC-K_2_CO_3_ and PANC-KNO_3_ adsorbents. Furthermore, literature survey shows that the ID/IG ratio is also linked with the CO2 uptake ability and the porous framework (Haghseresht et al., 1999). As mentioned the PANC-NaOH and PANC-KOH adsorbents having a bit higher ID/IG ratio and therefore show high CO_2_ adsorption capacities as compared to other activated adsorbents.

**Figure 3:**
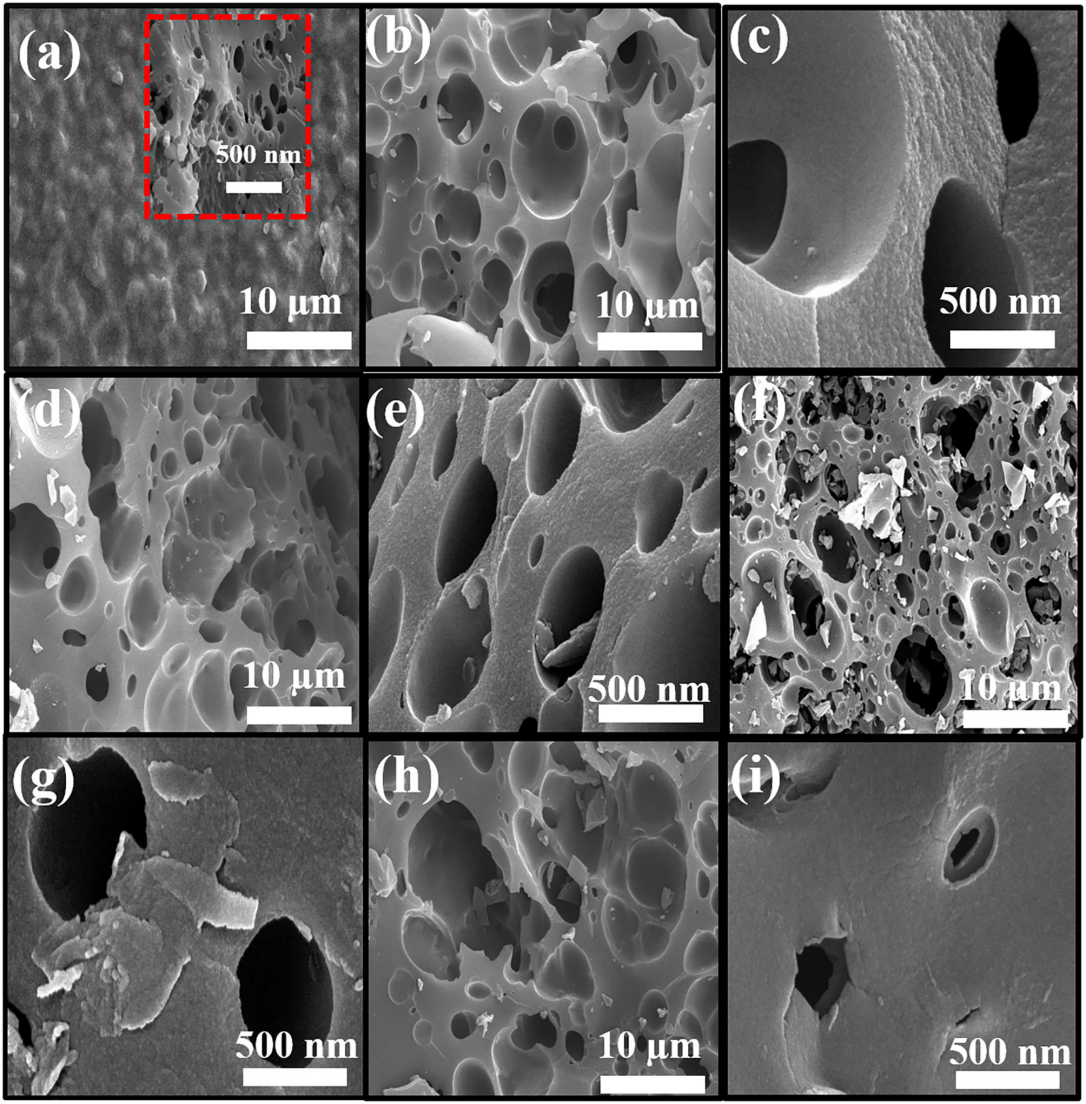
FE-SEM images of **(a)** PAN-C, **(b,c)** PANC-NaOH, **(d,e)** PANC-KOH, **(f,g)** PANC-K_2_CO_3_, and **(h,i)** PANC-KNO_3_ at various resolutions.

Figures 3a–i shows SEM images of PAN-C precursor and all of the activated carbon materials at various resolutions. As mentioned all the PAN-C activated materials shows a smooth surface with irregular morphology (Zhang et al., 2015). The SEM image of PAN-C precursor was obtained at 10 μm (Figure 3a) rough displays with no pores and defects. While the inset of Figure 3a recorded at 500 nm, shows the cracked surface with irregular interconnected network and few void spaces, that responsible for the very low specific surface area of PAN-C precursor. Whereas, the PAN-C activated material shown in Figures 3b–i clearly illustrates highly porous carbon materials. Small pits, defects and several pores were generated on the carbon surface because of the removal of volatile components during the chemical activation process with different activating agents that attribute to large specific surface area. The chemical activation of the PAN-C precursor led to the formation of precise pores with specific structures depending on the type of activator used.

**Figure 4:**
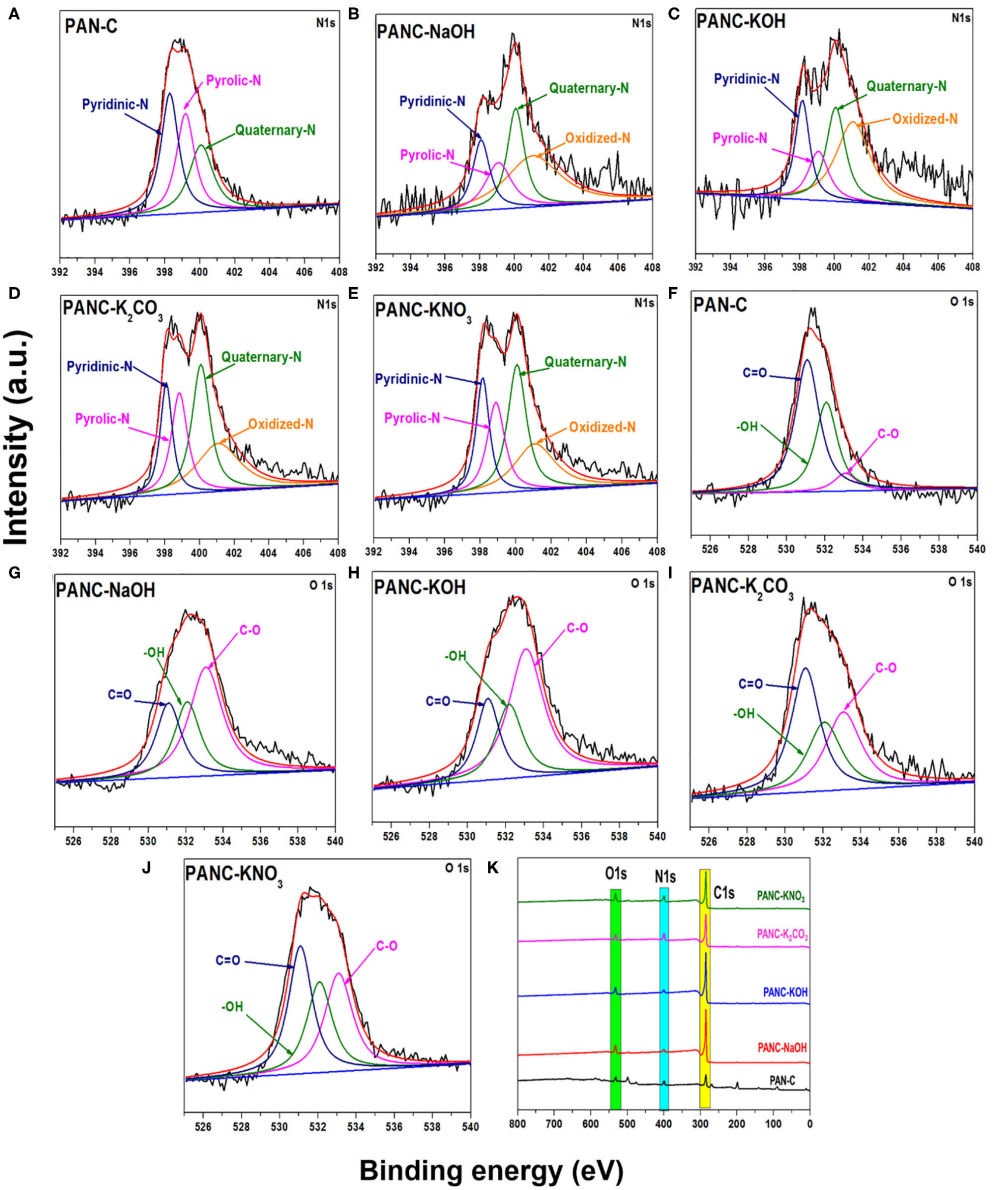
XPS N 1s scan of **(A)** PAN-C **(B)** PANC-NaOH **(C)** PANC-KOH **(D)** PANC-K_2_CO_3_ **(E)** PANC-KNO_3_ and O 1s scan of **(F)** PAN-C, **(G)** PANC-NaOH, **(H)** PANC-KOH, **(I)** PANC-K_2_CO_3_ and **(J)** PANC-KNO_3_, and **(K)** XPS survey analysis of all synthesized absorbents respectively.

Furthermore, the XPS analysis was performed to investigate the different nitrogen, carbon, and oxygen moieties exist above the surface of the all prepared porous carbon adsorbents (Figure 4). As mentioned in Figures 4A–E the deconvolution of 1Ns spectra represent four peaks with various binding energies indicating the existence of four types of nitrogen functionalities above the activated adsorbents surface, while the peak at 398.51 eV corresponds to pyridinic-N, peaks at 399.82 eV relates to pyrrolic-N/pyridone-N and signal at 400.08 and 401.07 eV attributed to Quaternary-N and Oxidized-N correspondingly (Sevilla et al., 2011; Tiwari et al., 2017). The peak signals of pyridinic-N in all activated carbon adsorbents are higher, which illustrates that the pyridinic-N functionality above the surface contributes more to increasing the CO_2_ uptake capacities (Lee and Park, 2013). This pyridinic-N functionality capable of increasing CO_2_ adsorption by providing the specific active sites for CO_2_ gas molecules (Hao et al., 2010). Beside this, the O1s spectra deconvulated into three major signals as mentioned in Figures 4F–J at 531.94, 532.6, and 533.07 eV attributed to oxygen functionality of carbonyl (C=O), hydroxyl (-OH) and C–O groups successively (Tiwari et al., 2017). Except PAN-C precursor, there were no additional peaks observed of potassium within the spectra of all activated carbon adsorbents which confirmed the complete elimination of metal potassium impurities within the porous adsorbents due to HCl rinsing (Figure 4K).

**Figure 5:**
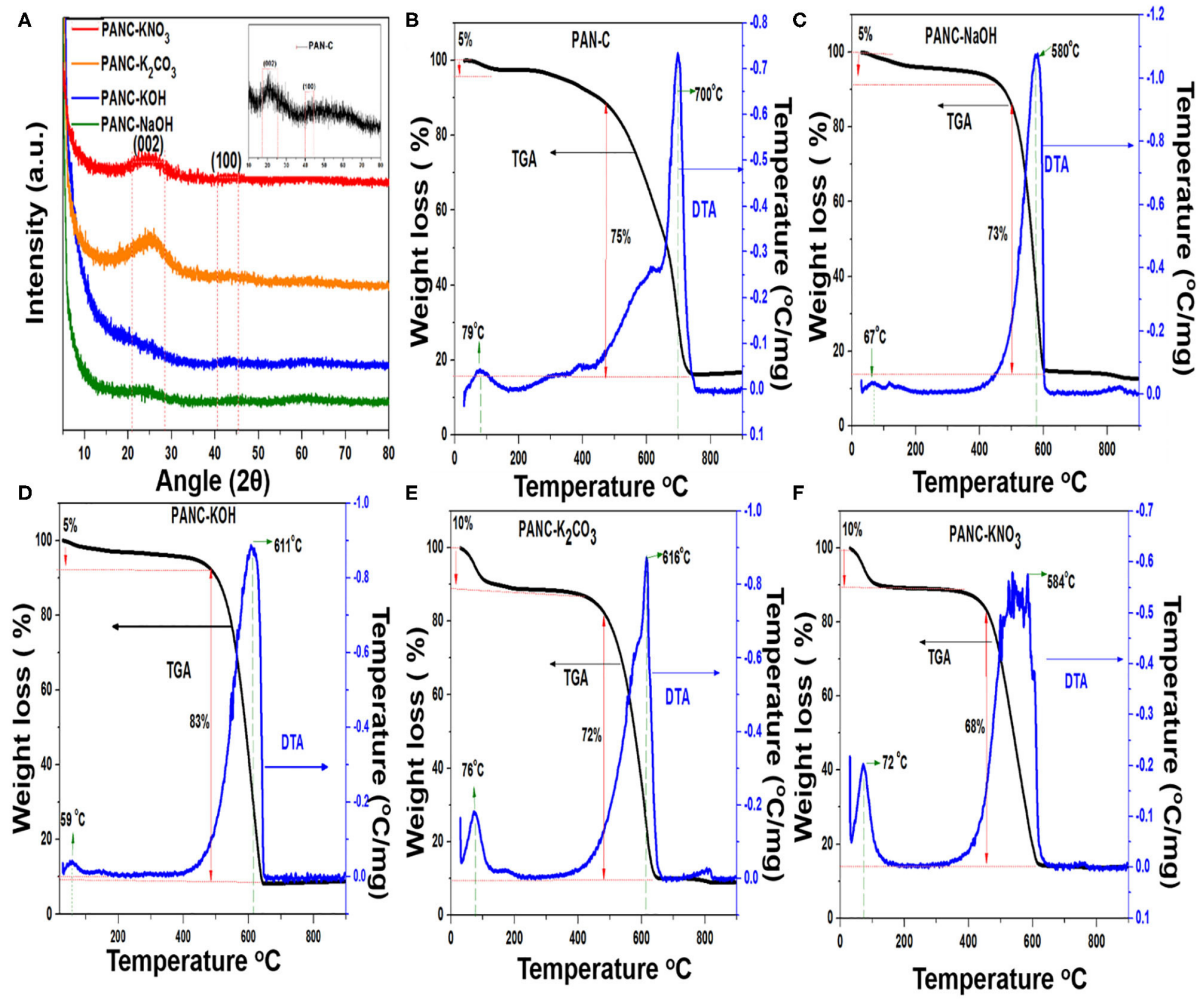
**(A)** XRD patterns of all activated materials and inset shows the XRD pattern of PAN-C sample and **(B–F)** TGA-DTA analysis of all prepared adsorbents.

Figure 5A, illustrates the XRD patterns of the PAN-C sample and all of the activated carbon materials. Each pattern shows two diffraction peaks at 25° and 43°, which are indexed to the (002) and (100) planes, respectively. These peaks indicate the presence of graphitic carbons. The (002) peaks correspond to a *d*-spacing of 3.43, which indicates a turbostratic arrangement of atoms within the graphitic layers (Liu et al., 2017).

The results of the TGA experiments reveal the thermal nature of the samples on the basis of their characteristic degradation phenomena. TGA in combination with differential thermal analysis (DTA) was conducted to obtain appropriate temperature ranges for the carbonization reactions as well as to elucidate the chemical activation mechanism. Figures 5B–F illustrates the TGA–DTA plot for PAN-C precursor and other samples activated with various activators. The TGA–DTA plots indicate that thermal degradation of all of the materials proceeded in two main steps: dehydration, followed by carbonization. The initial weight loss that occurred between 25 and 100°C because of water and moisture elimination from the pores within the adsorbents was 5% (in the case of PAN-C, PANC-NaOH and PANC-KOH) and 10% (for PANC-K_2_CO_3_ and PANC-KNO_3_); further confirmation was obtained from the initial endothermic peaks in the DTA plots. As shown in Figure 5B, a mass loss of 75% occurred between 430 and 730°C temperature range for sample PAN-C, whereas the other activated samples exhibited a slightly greater continuous mass loss (68–83%) with a lower final temperature range (460–644°C), probably because of the cyclization of PAN, oxidation and degradation of nitrogen- and oxygen-containing species within the activated adsorbents (Kim et al., 2009), that was confirmed by elemental analysis (Table 1). However, the highest degradation temperature for each material is clearly shown in the DTA plots, which are consistent with the TGA data. The order for the activated materials weight loss was PANC-KOH (83%) > PANC-NaOH = PANC-K_2_CO_3_ (around 73%) > PANC-KNO_3_ (68%). The greater weight loss of the activated samples as compared with that of the PAN-C precursor and PANC-KNO_3_ is attributed to the greater carbon contents existence, consistent with the elemental analysis results. The quantitative CHN analysis results (Table 1) confirm the presence of various elemental contents within the prepared materials. It was noticed that, the carbon content of PAN-C precursor was very low (70.47%) as compared to the other activated samples (ranged between 79 and 88%), because after chemical activation and carbonization, the increase in the degree of graphitization of activated carbons occurred (Singh et al., 2019). All of the activated adsorbents contained large amounts of carbon, which contributed to an increase in adsorption capacity of CO_2_ because of contributions of van der Waals forces (Singh and Kumar, 2016). The PAN-C precursor have greater nitrogen contents (11.65%), although their nitrogen concentration changes according to the activating agent used, as confirmed from the decomposition of nitrogen species during the chemical activation process. In addition, the nitrogen content within the other activated adsorbents was 3.14–5.91%, demonstrating the addition or degradation of different nitrogen moieties during the activation mechanism using different activators. Furthermore, the atomic percentage obtained by XPS technique of all synthesized samples are summarized in Table 1. The acquired XPS elemental results show resemblance with the data obtained from EA technique, signifying the quantities of nitrogen, carbon and oxygen contents above the surface exists in almost similar amounts as that in the bulk.

**Table 1:** Elemental and XPS analysis of all fabricated samples.

**Specimens**	**Elemental analysis (weight %)**			**XPS analysis (at %)**		
	**C^a^**	**N^b^**	**H^c^**	**C^a^**	**N^b^**	**O^d^**
PAN-C	70.47	11.65	2.87	70.38	11.54	14.98
PANC-NaOH	88.54	5.91	1.79	87.95	5.78	3.76
PANC-KOH	89.63	5.72	1.23	90.01	5.56	3.18
PANC-K_2_CO_3_	80.69	3.89	2.46	80.56	3.96	12.67
PANC-KNO_3_	79.50	3.14	2.15	80.03	3.48	13.35

a *Carbon contents*.

b *Nitrogen contents*.

c *Hydrogen contents*.

d *Oxygen contents*.

### Analysis of Textural Properties

**Figure 6:**
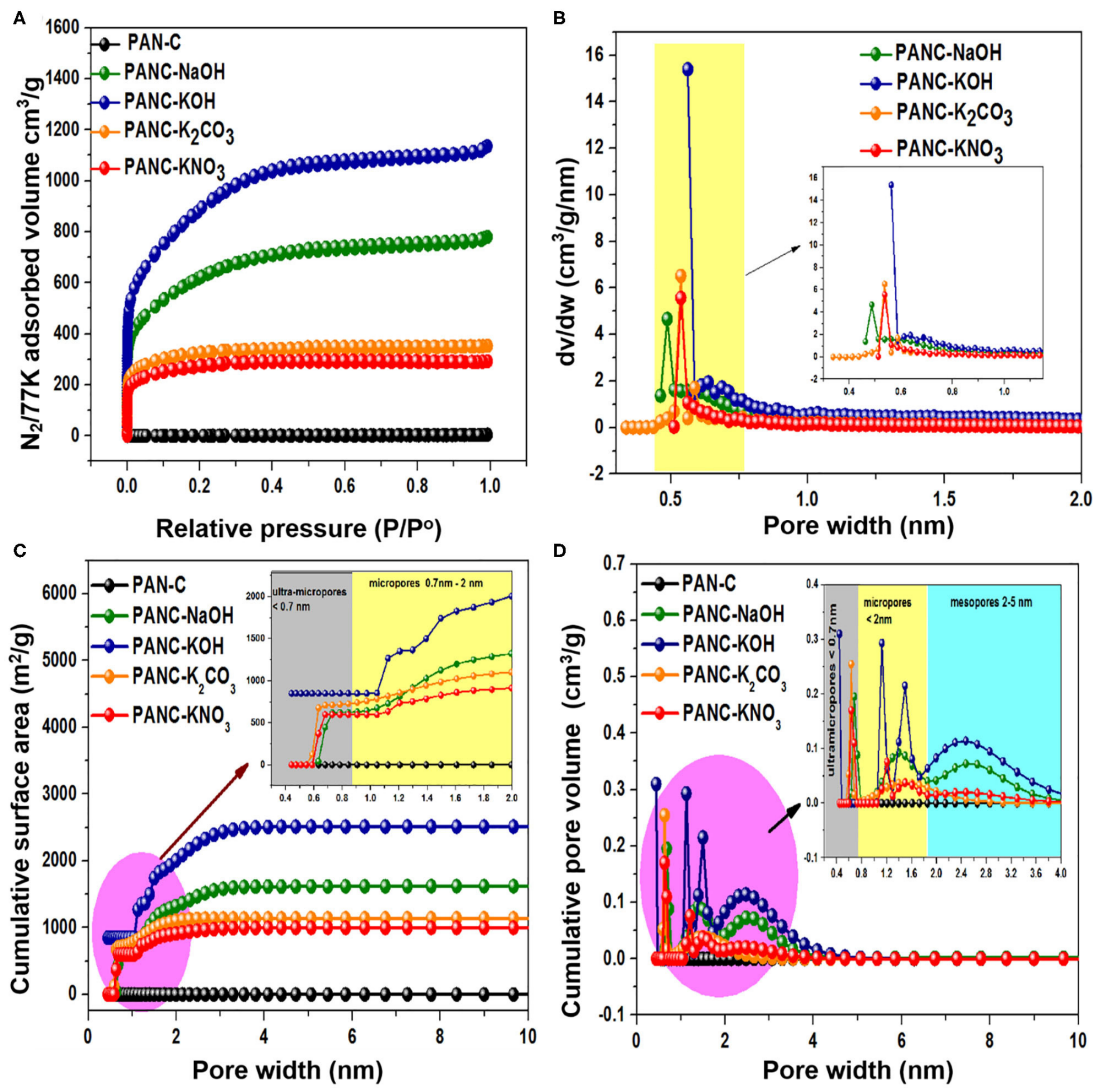
**(A)** N_2_ adsorption-desorption plots, **(B)** pore size distributions, **(C)** cumulative surface area, and **(D)** cumulative pore volume of all synthesized samples.

For the purpose of analyzing the process of pore formation by activators, nitrogen (N_2_) sorption isothermal data and cumulative surface area were collected at 77 K (Figures 6A,C), and the results were used to determine the respective pore size distributions and cumulative pore volume (Figures 6B,D). The textural properties of the as-synthesized samples are summarized in Table 2. Each selected activator with PAN-C precursor provided activated adsorbents with specific textural features including high specific surface area (971–3,072 m^2^ g^−1^) and high micropore volume (0.387–1.159 cm^3^ g^−1^). The PAN-C samples synthesized in the absence of an activating agent exhibited very low minimum surface areas; consequently, the BET measurement did not yield precise results. All the activated adsorbents show an intense increase in nitrogen uptakes at minimum relative pressure (<0.01), which represent extreme microporous nature of all synthesized activated adsorbents (Cychosz and Thommes, 2018). The activation with NaOH and KOH activators shows a significant increase in the N_2_ adsorption, demonstrating the generation of efficient textural porosities due to the decomposition and removal of gaseous species from the porous framework. The PANC-NaOH and PANC-KOH adsorbents exhibit two types of adsorption isotherms (Type I + Type IV) (Figure 6A), which is attributed to the formation of different types of pores sizes and this kind of isotherms are in agreement with the previously reported work (Shen et al., 2011). As compared to this, the N_2_ adsorption plots of adsorbents activated by K_2_CO_3_ and KNO_3_ activators show narrower Knee with type I isotherms. More precisely, it can have concluded that by varying the activators, the N_2_ adsorption amount automatically varied that lead to the tailoring in textural features (Singh et al., 2019). In Figure 6A, it was observed that in N_2_ adsorption isotherm of PANC-KOH and PANC-NaOH adsorbents, an increase in slop along with a shift in relative pressure (P/P°) from 0.10 to 0.4 demonstrating the widening of pores to form several mesopores and micropores (Silvestre-Albero et al., 2014). While, in PANC-K_2_CO_3_ and PANC-KNO_3_ isotherm, an intense rise in knee of adsorption isotherm occurs primarily and after reaching to 0.3 relative pressure, no substantial rise in N_2_ adsorption was observed. A surface of the heterogeneous nature of the prepared porous adsorbents is normally measured in relation to their pore size distributions. As shown in Figure 6B, the overall pore-size distribution of all of the activated adsorbents exists in the range of 0.4–0.8 nm. Interestingly, all the activated adsorbents display a significant fraction of micropores <1 nm, due to the observation of a large number of peaks within the region <0.8nm. The intense peaks centered at 0.48, 0.56, and 0.53 nm for PANC-NaOH, PANC-KOH, and PANC-K_2_CO_3_ = PANC-KNO_3_, respectively. Although the same intense peak position noticed for sample activated by K_2_CO_3_ and KNO_3_, but the concentration of micropores within PANC-K_2_CO_3_ adsorbents are more than that of PANC-KNO_3_ adsorbent. Overall, the specific surface area (SSA) exhibited the following order: PANC-KOH (3,072 m^2^ g^−1^) > PANC-NaOH (2,012 m^2^ g^−1^) > PANC-K_2_CO_3_ (1,179 m^2^ g^−1^) > PANC-KNO_3_ (971 m^2^ g^−1^). The corresponding total pore volume (V_total_) values were 1.75, 1.20, 0.54, and 0.45 cm^3^ g^−1^, respectively (Table 2). The NLDFT theory was used to determine the microporous structures. The largest micropore volume was that of PANC-KOH (1.159 cm^3^ g^−1^), which decreased to 0.387 cm^3^ g^−1^ (PANC-KNO_3_) because of different activating agents.

**Table 2:** Textural properties of fabricated adsorbents and Raman calculations.

**Specimens**	**SSA^a^ (m^2^/g)**	**Pore volume (cm^3^/g)**				**Raman calculation**		
		**V _<1 nm_^b^**	**V _<2 nm_^c^**	**V _<5 nm_^d^**	**D_pore_^e^ (nm)**	**D band (cm^−1^)**	**G band (cm^−1^)**	**I_D_/I_G_**
PAN-C	3	0.007	-	-	10.4	-	-	-
PANC-NaOH	2,012	1.204	0.825	0.379	2.4	1,325	1,588	1.03
PANC-KOH	3,072	1.753	1.159	0.594	2.3	1,320	1,607	1.04
PANC-K_2_CO_3_	1,179	0.545	0.465	0.080	1.9	1,331	1,590	0.96
PANC-KNO_3_	971	0.452	0.387	0.065	1.8	1,320	1,584	1.02

SSA^a^: *specific surface area computed by BET method*.

V _<1nm_^b^: *total pore volume obtained from the NLDFT method at P/P° = 0.98*.

V _<2nm_^c^: *micropore volume calculated by NLDFT method*.

V _<5nm_^d^: *Mesopore volume computed by NLDFT method*.

**D _pore_^e^:***pore diameters at the maxima of pore size distribution obtained by NLDFT method*.

Moreover, the cumulative surface area and cumulative pore volume of all the fabricated adsorbents are graphed vs. pore width (nm) as mentioned in Figures 6C,D. It was illustrated that, the cumulative surface area of PANC-KOH increases sharply in the region of 1–2 nm, while in other activated adsorbents, the increase in surface area was started from 0.7 nm (Figure 6C). From the plot of cumulative pore volume (Figure 6D), it has been observed that in PANC-NaOH and PANC-KOH adsorbents exhibited high concentration micropores (<2 nm) and mesopores (2–4 nm), as well as in ultra-micropores (<0.7 nm). But PANC-K_2_CO_3_ and PANC-KNO_3_ adsorbents shows more intense peaks in ultra-micropores region (<0.7 nm) as compared to micropores region (<2 nm) and almost negligible peaks were noticed in mesopores fraction. These consequences reveal that the CO_2_ uptakes ability is highly linked with the nature of existed porosities that was obtained by using different activating agents (Qian et al., 2014).

The maximum SSA and highest pore generation within the PANC-NaOH and PANC-KOH adsorbents are caused by gasification reactions among carbon and the hydroxide activators. The chemical activation is a single-stage mechanism involving the formation of activated carbons with well-synthesized pores. Equations (1–2) show the chemical transformation that occurs through the chemical-activation phenomena involving hydroxide activating agents (NaOH and KOH):

(1) $$\begin{array}{l} \text{C}+{\text{H}}_{2}\text{O}\to \text{CO}+{\text{H}}_{2} \end{array}$$

(2) $$\begin{array}{l} \text{C}+\text{C}{\text{O}}_{2}\to 2\text{CO} \end{array}$$

These equations show the chemical transformation of PAN-C precursor into activated adsorbents followed by the physical activation procedure involving CO_2_ gas (Linares-Solano et al., 2012). The generated CO_2_ can combine to destroy the carbon surface, leading to the development of pores and also promotes the formation of wider pores (Mistar et al., 2018).

The gasification reactions leading to the PANC-NaOH product are shown in Equations (3–6) (Lillo-Ródenas et al., 2003):

(3) $$\begin{array}{l} 4\text{NaOH}+\text{C}\to 4\text{Na}+\text{C}{\text{O}}_{2}+2{\text{H}}_{2}\text{O} \end{array}$$

(4) $$\begin{array}{l} 6\text{NaOH}+2\text{C}\to 2\text{Na}+3{\text{H}}_{2}+\text{N}{\text{a}}_{2}\text{C}{\text{O}}_{3} \end{array}$$

(5) $$\begin{array}{l} 4\text{NaOH}+2\text{C}{\text{O}}_{2}\to 2\text{N}{\text{a}}_{2}\text{C}{\text{O}}_{3}+2{\text{H}}_{2}\text{O} \end{array}$$

Overall,

(6) $$\begin{array}{l} 6\text{NaOH}+2\text{C}\to 2\text{Na}+3{\text{H}}_{2}+3\text{N}{\text{a}}_{2}\text{C}{\text{O}}_{3} \end{array}$$

In general, the NaOH activator responsible oxidizes and destroys the carbon material surface, thereby leading to an increase in the SSA; it is also responsible for the formation of pores with a larger diameter (2.39 nm) (Setyaningsih et al., 2008). Similarly, the greater SSA and porosity of the KOH-treated adsorbents are also attributable to the occurrence of the gasification reactions shown in Equations (7–12):

(7) $$\begin{array}{l} 2\text{KOH}\to {\text{K}}_{2}\text{O}+{\text{H}}_{2}\text{O} \end{array}$$

(8) $$\begin{array}{l} \text{C}+{\text{H}}_{2}\text{O}\to {\text{H}}_{2}+\text{CO} \end{array}$$

(9) $$\begin{array}{l} \text{CO}+{\text{H}}_{2}\text{O}\to {\text{H}}_{2}+\text{C}{\text{O}}_{2} \end{array}$$

(10) $$\begin{array}{l} {\text{K}}_{2}\text{O}+\text{C}{\text{O}}_{2}\to {\text{K}}_{2}\text{C}{\text{O}}_{3} \end{array}$$

(11) $$\begin{array}{l} {\text{K}}_{2}\text{C}{\text{O}}_{3}+2\text{C}\to 2\text{K}+3\text{CO} \end{array}$$

Overall,

(12) $$\begin{array}{l} 4\text{KOH}+\text{C}\to {\text{K}}_{2}\text{C}{\text{O}}_{3}+{\text{K}}_{2}\text{O}+2{\text{H}}_{2} \end{array}$$

At 800°C, KOH produces metallic K, which combines with the PAN-C carbon material to develop micropores and mesopores within the surface. Furthermore, the hydrochloric acid rinsing of prepared carbon material also contributed to the development of the highly porous surface because it eliminates residual metals (K, Na). The wide variation in the SSA between the PANC-KOH and PANC-NaOH and all of the other adsorbents demonstrates the effectiveness of NaOH and KOH for achieving better surface properties than KNO_3_ and K_2_CO_3_ activating agents. The efficiency of different activating agents in treating the PAN-C precursor follows the sequence KOH > NaOH > K_2_CO_3_ > KNO_3_. Accordingly, it can be resultant that activators contribute essentially in the improvements of textural properties of the materials.

### Gas Adsorption Studies

**Figure 7:**
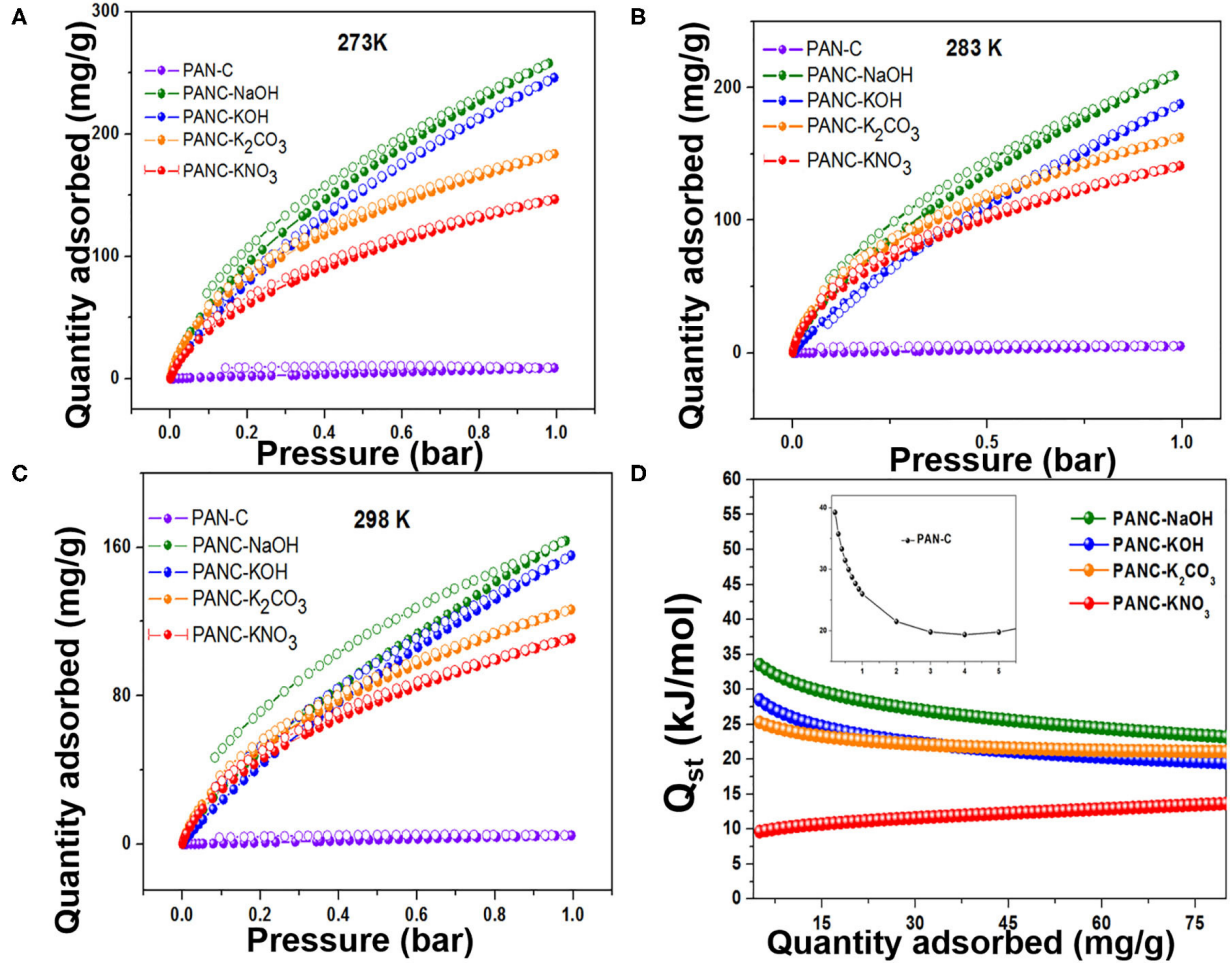
Adsorption-desorption isotherms of CO_2_ on all fabricated carbon materials at **(A)** 273 K, **(B)** 283 K and **(C)** 298 K, and **(D)** Isosteric heat of adsorptions of all as-synthesized materials.

The synthesized activated carbon adsorbents having heteroatoms (nitrogen and oxygen) contained carbon framework and exhibited extremely high SSAs and various microporous and mesoporous morphologies with different pore diameters. Because of these efficient nature of the prepared adsorbents, we investigated their CO_2_ adsorption behavior. As shown in Figures 7A–C, the CO_2_ adsorption–desorption experimental plots for all of the fabricated materials were carried out at three temperatures (273, 283, and 298 K) at 1 bar pressure; the overall adsorption values at the specified temperatures are summarized in Table 3. The effect of adsorption temperature on the gas uptake tendency of adsorbents is an important consideration. Because CO_2_ capture is an exothermic adsorption process, CO_2_ capture performance tends to decline with increasing adsorption temperature. The reason behind is the increase in molecular kinetic energy (K.E) of the CO_2_ molecules at higher temperature.

**Table 3:** CO_2_ and N_2_ adsorption measurement of all synthesized adsorbents.

**Specimens**	**CO_2_ uptake**			***Q_st_* (kJ/mol)**	**N_2_ uptakes (mg/g)**	
	**273 K (mg/g)**	**283 K (mg/g)**	**298 K (mg/g)**		**273 K**	**298 K**
PAN-C	8.95	5.17	4.63	39.26	-	-
PANC-NaOH	257.97	209.58	163.57	33.54	19.02	12.30
PANC-KOH	246.23	187.8	155.66	28.49	19.67	12.49
PANC-K_2_CO_3_	183.96	162.65	126.47	24.41	13.45	8.11
PANC-KNO_3_	146.89	141.14	111	10.81	12.08	7.08

The PANC-NaOH and PANC-KOH both exhibited high CO_2_ adsorption capacities (i.e., 163–257 mg g^−1^ (3.71–5.86 mmol g^−1^) and 155–246 mg g^−1^ (3.53–5.59 mmol g^−1^) compared with the other activated adsorbents (Table 3) at 273 and 298 K and at 1 bar, respectively. The high CO_2_ adsorption performance of PANC-NaOH and PANC-KOH is attributed to their high SSAs (2,012 and 3,072 m^2^ g^−1^) and large micropore volumes (0.82–1.15 cm^2^ g^−1^). Furthermore, the existence of nitrogen groups in the form of pyridinic-N also plays a vital role in increasing the adsorption performance of both activated carbons samples. As mentioned in Table 1, among all the activated carbon adsorbents the PANC-NaOH and PANC-KOH samples exhibits higher nitrogen contents (5.91 and 5.72%) which also considered a factor that contributes to maximum CO_2_ adsorption. The minor difference in adsorption capacities between PANC-NaOH and PANC-KOH is attributed to the slightly broader pore size distribution (1.75 cm^2^ g^−1^) of PANC-KOH. The greater SSA and micropore volume result in greater availability of physically activated sites for CO_2_ capture onto the adsorbents, which leads to greater adsorption by the activated adsorbents.

**Figure 8:**
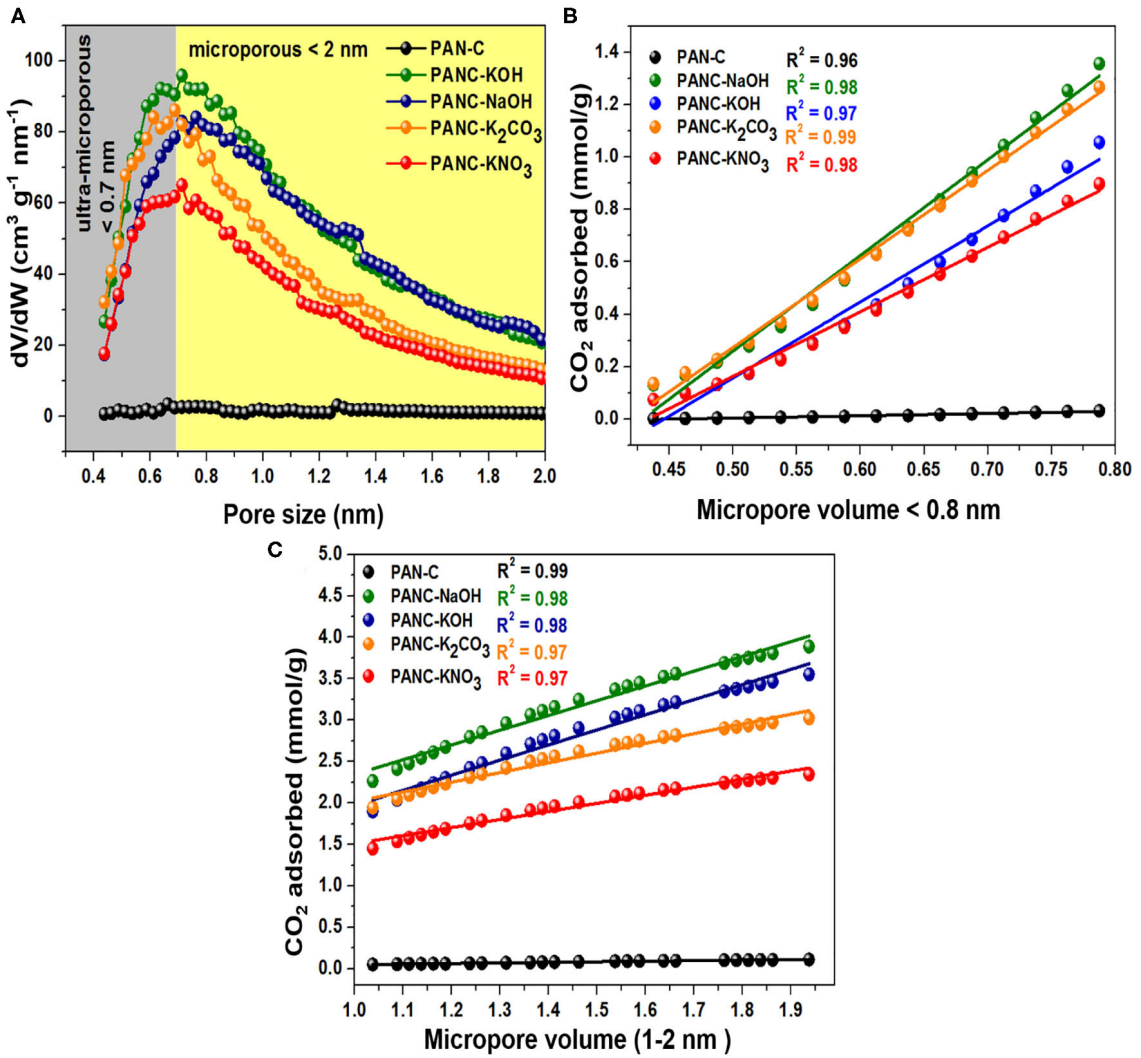
**(A)** Micropores size distributions carried out by HK calculations through CO_2_ adsorption data, correlation among CO_2_ uptakes and the fraction of micropore volume **(B)** < 0.8 nm and **(C)** between 1 and 2 nm, at 273 K and 1 bar.

Furthermore, the ultra-microporosities within carbon-based materials contribute more toward efficient CO_2_ adsorption. Typically, the N_2_ adsorption-desorption plots carried out at 77 K, provide information about textural features of adsorbents. But the CO_2_ adsorption isotherm that was calculated at 273 K and 1 bar seems very useful to study narrow micropores role because of the facile diffusion of CO_2_ molecules within the interlayers spacing covering microspores. The reason behind this diffusion of gas inside the micropores is the narrow kinetic width around 0.33 nm in contrast to N_2_ width that is equal to 0.36 nm (Sevilla et al., 2013). Moreover, as the temperature of CO_2_ adsorption increased leads to an increase in kinetic energy (K.E) of gaseous species. Resultantly, the declined an energy barrier for the CO_2_ uptakes inside the narrow micropores occurred (Sevilla et al., 2018). Herein, Horvath–Kawazoe (H–K) method was applied on the CO_2_ capture practical data of all activated carbon adsorbents that was carried out at 273 K/1 bar pressure, in order to illustrate the role of ultra-microporosities (<0.7 nm) and micropores (<2 nm) toward enhanced CO_2_ adsorption. Figure 8A, shows the pore size distribution calculated by H-K method (at 273 K/1 bar of CO_2_ gas) of all activated carbon adsorbents and it was observed that ultra-micropores (<0.7 nm) is seemed to possess effective adsorption active sites for CO_2_ molecule at <1 bar. At the initial stage, the ultra-micropores are capable of occupied with CO_2_ molecules because of their greater adsorption efficiency. By considering these textural properties of synthesized activated carbon adsorbents, it can have acknowledged that ultra-micropores (<0.7 nm) plays a very essential role in CO_2_ uptakes.

Additionally, for the purpose to elaborate the effect of textural characteristics (specifically microporosities), we correlate the CO_2_ adsorption data obtained at 273 K/1bar of all activated carbon adsorbents with the fraction of ultra-micropores <0.8 nm and with the fraction of micropores ranged within the 1–2 nm (Figures 8B,C). In Figure 8B, it was observed that CO_2_ uptakes rises continuously as the ultra-microporosity increases from 0.48 to 0.79 nm for all activated carbon adsorbents and exhibits a high correlation coefficient (*R*^2^) values (between 0.97 and 0.99) with the linear graph. While the CO_2_ adsorption within the ultra-micropore (<0.8 nm) of PAN-C precursor remains constant without any increase in CO_2_ adsorption capacities because of its poor microspores nature. As mentioned in Figure 8C, the straight-line graph was obtained within the micropore region of 1–2 nm for all activated carbon adsorbents with *R*^2^ values close to unity (0.97–0.99). Consequently, the micropores (<2 nm) also exhibited an essential role in improving CO_2_ adsorption performance. The obtained conclusions are in accordance with the previously studied work (Sevilla et al., 2011), they noticed that microporosity possess a vital role in describing the CO_2_ uptakes tendencies. The CO_2_ uptakes improved within activated carbon adsorbents due to the existence of ultra-micropores and micropores, that offer appropriate interaction for CO_2_ gas because of Vander Waal forces outside the micropores wall (Hong et al., 2016). Thus, the CO_2_ uptakes can be increased by the fabrication of adsorbents that contain ultra-microporosity (<0.8 nm) and micropores (<2 nm) by using various activators.

As it was noticed that the PAN-C precursor shows very low CO_2_ adsorption performance even though it exhibited higher nitrogen contents (11.65%) among other activated adsorbents, these nitrogen contents can help in increasing the interaction for CO_2_ molecules. But the negligible detectable surface area seems responsible for extremely minimum CO_2_ adsorption performance. Therefore, the conversion of PAN-C precursor into the carbonized adsorbents extensively raised the CO_2_ uptakes tendencies, usually because of improved textural characteristics; including SSA and pore size. Consequently, the pore size distribution also possesses crucial role in defining the adsorption performances (Yoo et al., 2013). The experimental data (Table 3) clearly show that the CO_2_ adsorption capacity of all of the samples decreased in the following order: PANC-NaOH > PANC-KOH > PANC-K_2_CO_3_ > PANC-KNO_3_. For comparison, Singh et al. (2019) performed similar CO_2_ adsorption experiments with KOH activated sample and reported a maximum adsorption capacity of 1.2 mmol g^−1^, which is lower than the capacity of our reported PANC-NaOH (3.71 mmol g^−1^) adsorbent at room temperature. Thus, the textural properties as well as existence of carbon, nitrogen and oxygen functional groups strongly influenced the CO_2_ adsorption performance of the adsorbents. The PANC-NaOH adsorbent, which is considered a prospective material for CO_2_ adsorption because of its highest CO_2_ uptake among the investigated adsorbents, can serve as a model adsorbent for future applications. Surprisingly, recent adsorption research has resulted in adsorbents with dominant performance over previously reported adsorbents, as summarized in Table 4.

**Table 4:** Comparative analysis of current research work for CO_2_ adsorption tendency and specific surface area at 273 K (1 bar) with the previously reported adsorbents.

**Materials**	**BET SSA (m_2_/g)**	**CO_2_ uptakes (273 K, 1 bar mg/g)**	**References**
PANC-NaOH	2,012	257	Present
PANC-KOH	3,072	246	Present
CF-850-act	1,018	125	Heo and Park, 2015
PAN-PK	2,231	194	Shen et al., 2011
NEPB-3UK	982	212	Singh et al., 2018b
AC-800-1	2,448	222	Zhang et al., 2013
NHPC4	1,150	174	Bing et al., 2017
APAB-3	2,232	181	Singh et al., 2018a
PR4_700	1,017	225	Liu et al., 2015
PAF-1-450	1,191	198	Ben et al., 2012
HCMDAH-1900-1	1,392	215	Hao et al., 2011
y-POP-A3	84	85.8	Kong et al., 2019

Moreover, to evaluate the nature of contact among CO_2_ molecules and the surface of the sorbents and to identify the adsorption phenomena, we used the Clausius–Clapeyron equation to model the experimental adsorption–desorption isothermal data recorded at 273 and 298 K to determine the isosteric heat of adsorption (Δ*Q*_st_) (Pan et al., 1998):

$$\begin{array}{l} Q_{st}=R\left[\partial \text{ln}\frac{P}{\partial \left(\frac{1}{T}\right)}\right]\theta \end{array}$$

where P is the pressure, R is the ideal gas constant, T is the sorption temperature range and θ is the number of adsorbed sites. As shown in Figure 7D and Table 3, the initial *Q*_*st*_ values of all of the adsorbents were in the range 10.81–39.26 kJ mol^−1^, which is typical for physisorption by carbon-based materials. It has been observed that PAN-C precursor exhibited high initial *Q*_*st*_ value (39.2 kJ mol^−1^) as comparted to other activated adsorbents, due to the highest availability of nitrogen contents which provides an excessive active binding sites for CO_2_ molecules that leads to the strong interaction between CO_2_ molecules and adsorbent surface. The isosteric heat of adsorption for PANC-NaOH is also reasonably high (33.12 kJ mol^−1^), indicating greater electrostatic interaction between acidic CO_2_ molecules and basic nitrogen functional sites on the adsorbents. Further involvement of the isosteric heats of adsorption arises from non-electrostatic interactive forces that exist within the micropores of the adsorbents. The increase in *Q*_*st*_ values with increasing CO_2_ adsorption reflects greater surface coverage, demonstrating the surface heterogeneity and developed surface chemistry of the synthesized adsorbents. These determined *Q*_*st*_ values are higher than previously reported results (28.4–10.5 kJ mol^−1^) (Hsu et al., 2010), suggesting a comparatively stronger attraction toward CO_2_ molecules.

**Figure 9:**
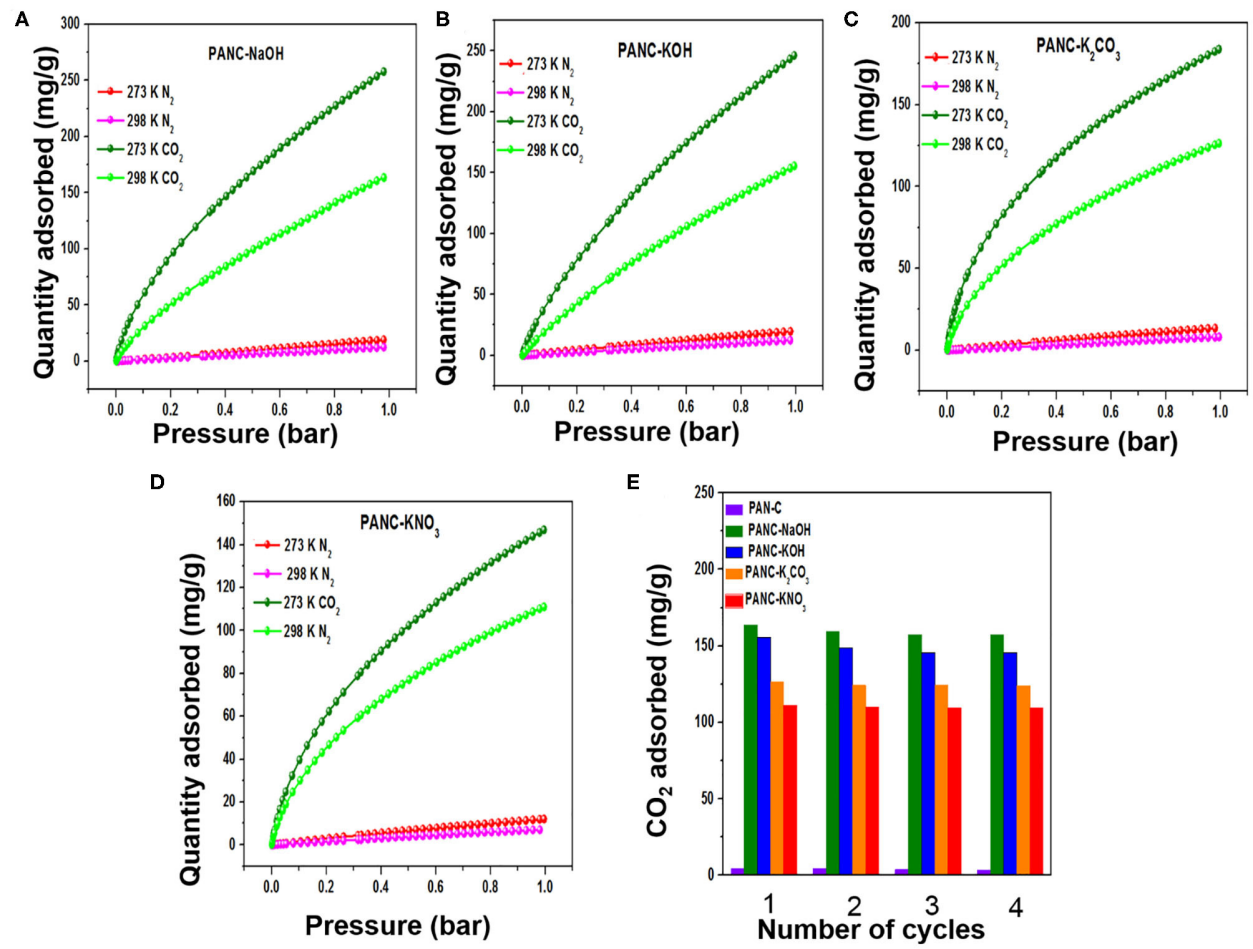
**(A–D)** CO_2_/N_2_ adsorption plots of all activated adsorbents for gas selectivity studies, and **(E)** repetitive cyclic adsorption measurements of all synthesized adsorbents at 298 K/1bar.

During the selection of potential adsorbents with the highest CO_2_ uptake, the selective behavior of adsorbents for CO_2_ gas over the other gases (i.e., N_2_, H_2_, and CH_4_) should be considered. If an adsorbent exhibits a tendency to capture all of the introduced gases simultaneously, then the adsorbent is an inefficient CO_2_ adsorbent. To investigate the selective adsorption performance of the synthesized adsorbents, we compared the CO_2_ and N_2_ adsorption data collected at 273 and 298 K (1 bar), as shown in Figures 9A–D and summarized in Table 3. In both temperature ranges, the adsorbents exhibit extremely high CO_2_ adsorption tendencies compared with N_2_. This result is attributed to the superior uptake of CO_2_ as a consequence of its high polarizability (29.11 × 10^−25^ cm^−3^) and intense quadrupole moment (4.30 × 10^−26^ esu^−1^ cm^−1^) compared with those of N_2_ (17.40 × 10–25 cm^−3^ and 1.52 × 10^−26^ esu^−1^ cm^−1^, respectively) (Balasubramanian and Chowdhury, 2015; Chowdhury et al., 2015). The CO_2_/N_2_ selectivity of activated adsorbents was determined by Henry's law and is represented as follows:

$$\begin{array}{l} S_{x,y}=\frac{K_{x}}{K_{Y}} \end{array}$$

This indicates the adsorption selective nature for CO_2_ gas (x) over N_2_ gas (y) respectively.

The determined adsorption selectivity values at 273 K and 1 bar were 27.7 (PANC-NaOH), 17.6 (PANC-KOH), 28.1 (PANC-K_2_CO_3_), and 21.2 (PANC-KNO_3_) for CO_2_ over N_2_. The calculated selectivity values at 298 K and 1 bar were 18.4 (PANC-NaOH), 15.8 (PANC-KOH), 33.6 (PANC-K_2_CO_3_), and 28.2 (PANC-KNO_3_). Therefore, the synthesized adsorbents with various activators are considered as potential candidates for efficient selective CO_2_ adsorption.

The recyclability of the all of the synthesized carbon materials should also be considered from an economic perspective. In the current research, we evaluated this feature of adsorbents by conducting four consecutive CO_2_ adsorption–desorption measurements at 298 K (1 bar), as mentioned in Figure 9E. The data clearly demonstrate that all of the synthesized adsorbents exhibited good regeneration ability; we therefore concluded that the fabricated carbon adsorbents are candidates for use in clean eco-friendly applications as well as for gas storage purposes.

## Conclusion

In this work, we designed high-SSA activated porous carbons with hetero-atom moieties from PAN-C precursor and synthesized them via a chemical activation method followed by carbonization to characterize their performance as CO_2_ adsorbents. After the chemical activation process of the PAN-C, the fabricated adsorbents exhibited an intensive increase in their surface structure as well as in their specific surface area and pore size distribution. The sorption progression was followed by physisorption. Briefly, PANC-NaOH, and PANC-KOH adsorbents with large surface areas exhibited CO_2_ adsorption capacities, with values 257 and 246 mg g^−1^ at 273 K and 163 and 155 mg g^−1^ at 298 K/1 bar, respectively. Furthermore, all of the activated adsorbents exhibited excellent CO_2_/N_2_ adsorption selectivity. The highest heat of adsorption (*Q*_*st*_) exhibited by PANC-NaOH (33.54 kJ mol^−1^) indicated the heterogeneous nature of the adsorbent surface, which exhibits maximum electrostatic and non-electrostatic interaction among CO_2_ molecules and adsorbents. In addition, all of the synthesized adsorbents exhibited efficient regeneration ability over four continuous adsorptions–desorption cycles, which confirmed that the prepared carbon adsorbents should be considered for CO_2_ capture in future applications.

## Data Availability Statement

The raw data supporting the conclusions of this article will be made available by the authors, without undue reservation.

## Author Contributions

UK designed the research idea, carried out the experiment, and wrote the manuscript. JC helped in reviewing and editing the manuscript. S-JP supervised and supported the project and also helped in writing, reviewing, and editing the manuscript. All authors contributed to the article and approved the submitted version.

## Conflict of Interest

JC was employed by the company Evertech Enterprise Co. Ltd. The remaining authors declare that the research was conducted in the absence of any commercial or financial relationships that could be construed as a potential conflict of interest.
